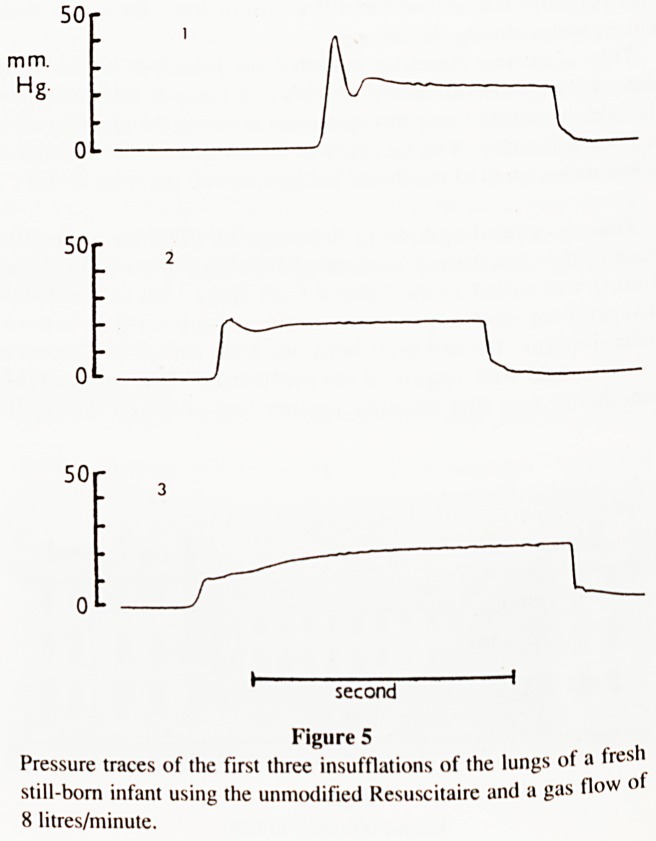# The Water-Ram of Bristol and Neonatal Insufflation

**Published:** 1991-03

**Authors:** Peter M Dunn

**Affiliations:** Professor of Perinatal Medicine and Child Health, University of Bristol, Southmead Hospital, Bristol


West of England Medical Journal Volume 106 (i) March 1991

### The Water-Ram of Bristol and Neonatal Insufflation

**Peter M Dunn, MD, FRCP, FRCOG**
Professor of Perinatal Medicine and Child Health,
University of Bristol, Southmead Hospital, Bristol.

When I was a young lad in the 1930s, our family used to spend the summer holidays at a cottage in Milton Eonan, Glenlyon, Perthshire. There was no electricity and water was pumped up into the cottage from the Milton Burn by a contraption known as the Ram. Powered by the gravity flow of unlimited water from the burn above, it maintained an intermittent jet of water into the attic tank; or rather it did until it suffered one of its periodic cardiac arrests. Although the Ram was 100 yards away, the sound of its beat was transmitted along the pipe and could be clearly heard in the cottage. Likewise, we always knew when it had stopped and required to be prodded once more into action, using a technique I later appreciated to be very similar to that used for external cardiac massage. My sisters and I took it in turns to administer the necessary resuscitation. Only if one could get back into the cottage without its re-arresting, could one claim to have completed one's turn. We used to hare back!

Thirty years later, these childhood memories were revived. I was sitting in the stack room of the University of Bristol Medical School library reading Munroe Smith's History of the Bristol Royal Infirmary[1] (Fig. 1) when I came across the following passage:

"During the alterations which were made this year (1797) a circumstance occurred which ought to be recorded.

A plumber was employed to fix a leaden pipe to carry water from a cistern on the middle storey of the building to the kitchen below. He found that when the tap at the end of this pipe was turned off, the sudden pressure of the long column of water above nearly always burst the pipe. To remedy this he soldered a smaller pipe immediately behind the tap and carried it to the same height as the cistern. This plan succeeded, and prevented the main pipe from bursting (Fig. 2).

It was noticed that when the tap was turned off a jet of water was ejected to a great height from the upper end of the smaller pipe. This additional pipe was therefore continued to the top of the building, and was utilised to fill a cistern from the waste water forced up by closing the tap.

This workman therefore invented the principle of the Water Ram. Montgolfier improved on this and made it self-acting, but the honour of first using this apparatus is due to the plumber at the Bristol Infirmary. The incident is mentioned in the Journal of Sciences and Arts of the Royal Institution, vol. ii., 3rd edition."

There is a third episode to the story. In 1973, ten years after reading that fascinating account of the discovery of the Water-Ram, I was called to the Special Care Baby Unit at Southmead Hospital to see a pre-term male infant with a tension pneumothorax. He had been born one hour earlier by Caesarean Section and had required resuscitation. The inescapable conclusion was that alveolar rupture had occurred during the

**Figure 1**
Bristol Infirmary in 1781

**Figure 2**
Plumbing modifications at the Bristol Infirmary in 1797.[1]

pulmonary insufflation at birth. In collaboration with two colleagues, Dr. Brian Speidel and Dr. Harry Perez-Alzueta, I decided that we must investigate the safety of our resuscitation equipment. This we did and reported our findings to the Paediatric Research Society the following year.[2] In short, all the six methods being used by us, and by others, for neonatal insufflation proved to be either ineffective or dangerous, and all our resuscitation apparatus had to be either discarded or modified. At this time, though, I will confine myself just to discussing the method that had been used on our pre-term infant with the pneumothorax.

The technique is illustrated in Fig. 3. There is a source of oxygen under pressure, a flowmeter and a tube leading to the baby with a T-piece underwater safety valve normally set to blow off at pressures over 30cm $H_20$ or 22mm Hg (Fig. 3). This is below the pressure usually considered to be capable of causing alveolar rupture. Positive pressure is provided by intermittently occluding the hole near the endotracheal tube. Usually the flowmeter is marked in litres per minute. However, unfortunately, the 'Resuscitaire' flowmeter that we were using was only marked in arbitrary flow units 1, 2 and 3. As we discovered during our investigations, each arbitrary unit was approximately equal to a flow of 3–4 litres/minute rather than the one litre/minute we had incorrectly assumed. The apparatus actually permitted a maximum flow of 10–12 litres/minute instead of the 1–3 litres/minute which would normally be regarded as quite adequate and safe.

Our next step was to make dynamic pressure recordings during insufflation. Pressure in the system was recorded on a polygraph using a Bell and Howell transducer and amplifier. As the lungs of fresh stillbirths were only occasionally available, we used instead for most of our observations an artificial rubber 'lung' with a maximal capacity of 60ml and a compliance of 4ml/cm $H_2O$ (0–30ml), which is approximately equivalent to the compliance of the newborn lung at 24 hours of age. Fig. 4A shows a typical trace of a single insufflation. However, most neonatal insufflation takes place at birth when the lungs are airless, though they may contain a lot of fluid, especially after Caesarean delivery. In these

**Figure 3**
Diagram of apparatus used for neonatal insufflation (see text).

**Figure 4**
Pressure traces of single insufflations using the 'Resuscitaire', an artificial lung, and an oxygen flow of 8 litres/minute.

A: Lung compliance: 4ml/cm $H_2O$ (0–30ml)
B: Lung compliance: Nil
C: Lung compliance: Nil but with inclusion of a dead-weight valve (see text).

circumstances lung compliance is nil. To mimic that situation we modified the rubber 'lung' by greatly reducing its size. When we insufflated the artificial lung with nil compliance, using the same flow rate, we noted a sharp initial peak pressure of 68mm Hg preceding the plateau (Fig. 4B). When I saw this I immediately thought of the Bristol plumber and his Water-Ram. The peak was undoubtedly due to a build-up of pressure in the system during the momentary inertial delay while the water column was being pushed down the manometer T-tube. Although the peak pressure was only maintained for less than one-tenth of a second, at the high flow-rate being used, the volume of gas delivered during this time was quite sufficient to rupture the non-aerated lungs of a newborn baby. While appreciating the somewhat different physical principles involved, we christened our observation the Water-Ram effect.

Next, we repeated our studies using the lungs from a still-born infant that had died just before delivery at term. The flow-rate was again 8 litres/minute. The pressure recordings of the first three insufflations are shown in Fig. 5. Notice the presence of the Water-Ram effect during the first insufflation. In the second insufflation the effect is smaller, while in the third, with the lung expanded, it has almost disappeared. Although the pressure in the system had only risen briefly to a height of 40mm Hg (55cm $H_2O$) during the first insufflation, we observed the formation of interstitial emphysema on the surface of the lungs and a steady bubble of gas through an underwater rupture of the pleura.

As a result of our experience, all 'Resuscitaires' throughout the country were modified by including in the circuit a dead-weight valve that blew off instantaneously at 40cm. $H_2O$ (30mm Hg). The trace shown in Fig. 4C was made using the modified apparatus and the same flow rate as previously. There was no Water-Ram effect, but just a plateau at 40cm. $H_2O$ with a series of small spikes due to the vibration of the dead-weight valve.

Thus, in summary, by a curious route the combination of childhood memories and the observation of a plumber at the Bristol Infirmary 200 years ago helped to lead to the exposure of a dangerous fault in apparatus designed to resuscitate newborn babies at birth.

### REFERENCES

1. SMITH, G.M. A History of the Bristol Royal Infirmary. Publ. by J.W. Arrowsmith, Bristol, 1917, pp 151–2.
2. DUNN, P.M., PEREZ-ALZEUTA, H. and SPEIDEL, B.D. Pitfalls in neonatal insufflation. *Arch Dis Childh*, **49,** 748, 1974.

**Figure 5**
Pressure traces of the first three insufflations of the lungs of a fresh still-born infant using the unmodified Resuscitaire and a gas flow of 8 litres/minute.

## Figures and Tables

**Figure 1 f1:**
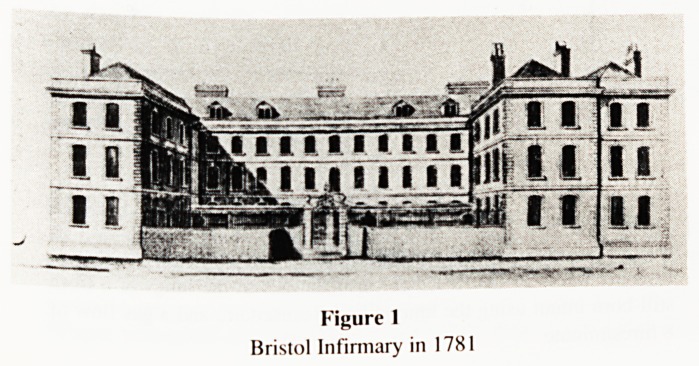


**Figure 2 f2:**
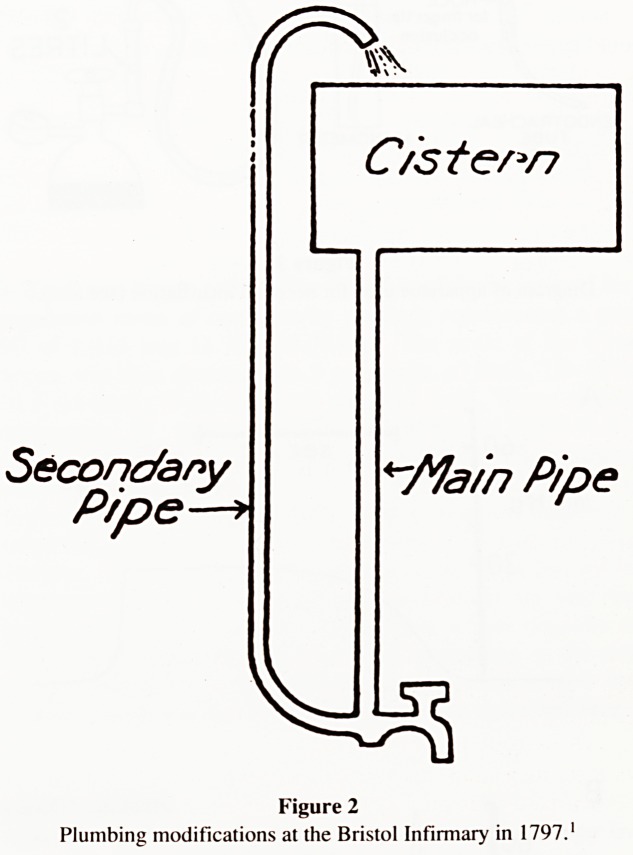


**Figure 3 f3:**
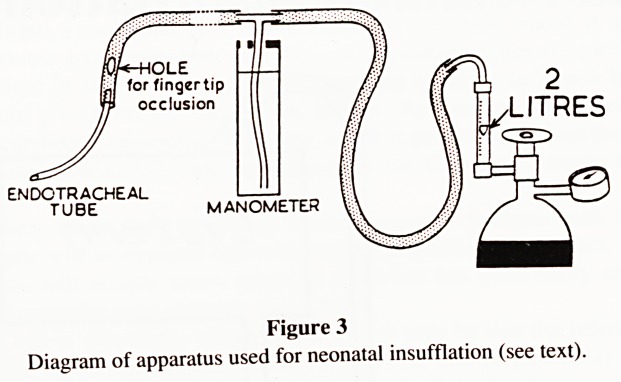


**Figure 4 f4:**
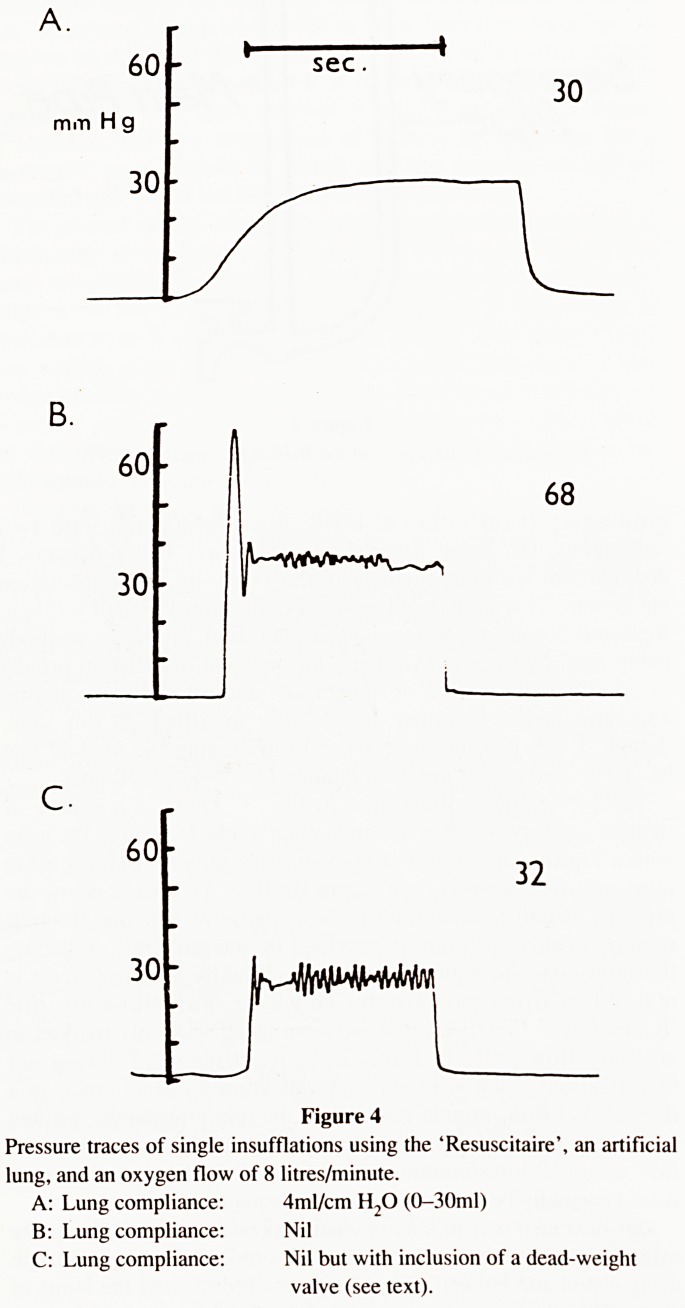


**Figure 5 f5:**